# Residue and Dissipation Kinetics of Metsulfuron-Methyl Herbicide in Soil: A Field Assessment at an Oil Palm Plantation

**DOI:** 10.3390/biom10071067

**Published:** 2020-07-16

**Authors:** Zainol Maznah, B. Sahid Ismail, Oii Kok Eng

**Affiliations:** 1 Malaysian Palm Oil Board, 6, Persiaran Institusi, Bandar Baru Bangi, Kajang 43000, Selangor, Malaysia; 2 School of Environmental and Natural Resource Sciences, Faculty of Science and Technology, Universiti Kebangsaan Malaysia, Bangi 43600, Selangor, Malaysia; ismailsahid@gmail.com (B.S.I.); sempoi_5576@yahoo.com (O.K.E.)

**Keywords:** metsulfuron-methyl, degradation, oil palm plantation, field study

## Abstract

A field trial experiment was conducted to investigate the degradation of metsulfuron-methyl at two application dosages, 15 g a.i/ha and 30 g a.i/ha, at an oil palm plantation. Soil samples were collected at ‒1, 0, 1, 3, 7, 14, and 21 days after treatment (DAT) at the following depths: 0–10, 10–20, 20–30, 30–40, and 40–50 cm. The results showed rapid degradation of metsulfuron-methyl in the soil, with calculated half-life (*t*_½_) values ranging from 6.3 and 7.9 days. The rates of degradation of metsulfuron-methyl followed first-order reaction kinetics (*R*^2^ = 0.91–0.92). At the spray dosage of 15 g a.i/ha, metsulfuron-methyl residue was detected at up to 20–30 cm soil depth, at 3.56% to 1.78% at 3 and 7 DAT, respectively. Doubling the dosage to 30 g a.i/ha increased the metsulfuron-methyl residue in up to 30–40 cm soil depth at 3, 7, and 14 DAT, with concentrations ranging from 1.90% to 1.74%. These findings suggest that metsulfuron-methyl has a low impact on the accumulation of the residues in the soil at application dosages of 15 g a.i/ha and 30 g a.i/ha, due to rapid degradation, and the half-life was found to be 6.3 to 7.9 days.

## 1. Introduction

Metsulfuron-methyl is a low-use-rate sulfonylurea herbicide widely used for post-emergence broadleaf weed control in oil palm plantations. It has good tolerance towards broadleaf weed and is very effective against a wide range of broadleaf weeds at application rates of 15 to 30 g a.i/ha [1,2]. Metsulfuron-methyl is a weak acidic compound with a pKa value of 3.3, and solubility in water varying with pH and ranging from 1100 to 9500 mg/L [3]. Degradation of sulfonylurea herbicides in soil is directly related to pH-dependent hydrolysis of the sulfonylurea bridge [4]. Once metsulfuron-methyl reaches the soil, it can be mineralized by microbial activity [5,6,7]. Mineralization is a process to describe a complete breakdown of an organic compound to CO_2_, H_2_O, and other simple inorganic compounds [8]. The major routes of metsulfuron-methyl mineralization in the environment are via the cleavage of the sulfonylurea bridge, O-demethylation of the methoxy–triazine moiety, and triazine ring opening after O-demethylation [8,9]. It has been reported that both metsulfuron-methyl hydrolysis and biological transformations were highly dependent on the soil temperature and moisture [9,10,11]. Furthermore, soil pH also plays a significant factor in affecting both sorption behaviour and chemical degradation of metsulfuron-methyl in the soil, due to its ability to influence the ionization state of the herbicide [9,12,13,14].

In the acidic soil, metsulfuron-methyl exists primarily in a non-anionic form in solution, which means it has better soil adsorption than in neutral and alkaline soils. Previous studies have reported that a highly significant negative correlation existed between metsulfuron-methyl adsorption and soil pH, because of greater phytotoxicity at a higher soil pH and low adsorption of the herbicide [15,16,17,18]. The literature reported that the extremely low levels of metsulfuron-methyl residues showed phytotoxic effects on many crops in crop-rotation systems [19]. Moreover, the phytotoxicity of metsulfuron-methyl is not only influenced by soil texture [20], but also decreases when the organic matter content in the soil was higher [18,19,20].

The degradation and leaching potential of metsulfuron-methyl have been extensively studied in various types of soils [21,22,23]. The increased use of metsulfuron-methyl has triggered concerns over the possibility of contamination by the herbicide, particularly its leaching potential to ground water. Recent study has shown that under simulated rainfall, metsulfuron-methyl residue can be observed at 30–35 cm column depth and is present in the leachate after seven days of application [23]. Based on the *K_OC_* (soil adsorption coefficient) values for a number of Argentinean soils, Azcarate et al. [21] found that the half-life (*t*_1/2_) for metsulfuron-methyl was more than 3 to 15 days, and that of sulfometuron-methyl was more than 3 to 24 days, which would classify them as the leachers in all soil horizons.

Previously, there h been a number of studies on the persistence, mobility, and bioactivity of metsulfuron-methyl conducted in Malaysian agricultural soils [4,24,25]. However, the information obtained did not resemble the actual situation and variables in the environmental factors involved. Most of the studies were conducted under laboratory conditions with controlled parameters [5,24,26]. When a pesticide is applied to a crop or soil, it will enter a dynamic ecosystem where it can migrate from one part of the system to another (e.g., uptake by plant from soil), degrade in situ, or move out of the system into other systems [27]. Even though a lot of studies have been carried out to study the behaviour and fate of pesticides in an oil palm plantation [28,29,30,31,32], only limited studies have reported on the behaviour of metsulfuron-methyl in the fields of oil palm plantation. Such studies are useful and needed to fill the voids resulting from laboratory findings. Therefore, the present study was initiated to provide quantitative information regarding the dissipation of metsulfuron-methyl under actual field conditions at oil palm plantation. The findings will provide a more holistic scenario for understanding the fate of metsulfuron-methyl in soil under natural conditions. It also gives more information about the potential risk of metsulfuron-methyl in the soil of a palm agro-ecosystem.

## 2. Materials and Methods 

### 2.1. Field Trial Experiment

The field trials were conducted at Sungai Buloh Oil Palm Estate, Selangor. The trial area consisted of 136 oil palm (DxP) trees within the age of 12 years old. The planted area is located on a flat coastal plain where the soil is predominantly clay loam. The plot size consisted of 16 rows with 12 palms per row, occupying about 1.5 ha total. Each plot was separated from the adjacent plots on all sides by 6 rows, to minimise lateral herbicide movement from one plot to another [4]. 

The field trial experiments were conducted using a randomised block design and divided into nine sub-plots to accommodate three treatments, namely metsulfuron-methyl (ALLY 20DF) at the rate of 15 g a.i/ha (1× the field rate), 30 g a.i/ha (2× the field rate), and the control (without applying any treatment). Spray applications of metsulfuron-methyl were carried out using a PB-16 knapsack sprayer fitted with the red multi-cone nozzle to deliver a volume rate of 450 L per hectare. The pressure was regulated at 1.5 to 2.0 bar. Treatments were applied over a 2.0 m wide swath in the harvesting paths and 1.8 m radius palm circles. 

### 2.2. Soil Sampling

Soil samples were collected at different ranges of depth; 0–10, 10–20, 20–30, 30–40, and 40–50 cm (using a soil auger) at 0, 1, 3, 7, 14, and 21 days after treatment (DAT). Five soil samples of approximately 0.5 kg each were randomly collected from every replicate across the study area. Soil samples collected from each replicate at the same depths were well-mixed and composited before being stored at −20 °C if not extracted immediately and analyzed within two weeks of storage. Prior to analysis, the soil samples were air-dried at room temperature and passed through a 2 mm sieve. Table 1 shows the soil characteristics, as determined at the Harris Laboratories, Inc. (Lincoln, NE, United States).

### 2.3. Determination of Metsulfuron-Methyl Residue 

The soil samples were analysed using a simple, modified extraction method proposed by Walker et al. [18]. Forty grams of sub-samples of the sieved soils were weighed into 250 mL centrifuge bottles. Residues of metsulfuron-methyl in the soil samples were extracted with 50 mL mixtures of methanol and water (70:30 *v/v*) containing 0.5% acetic acid (*v/v*) by shaking on a wrist-action shaker (Stuart, England) for 1 hour. Then, the extracts were separated from the soil by centrifugation at 11,000 rpm using a J-21C centrifuge (Beckman, United States). The supernatant was filtered through a 0.45 µm syringe filter before being injected into the HLPC-UV.

### 2.4. HPLC Conditions

Quantification of metsulfuron-methyl residue were performed using an HPLC equipped with an automated gradient controller, Waters 745 Data Module, Lambda-Max Model 481 UV detector, and Waters 501 pumps (Waters Scientific, Mississauga, ON, Canada). The analytical column used was a Jones Genesis reserve C18 column (15 cm × 4.6 mm). The mobile phase was acidified methanol (1% acetic acid) with MiliQ water (60:40, *v/v*). The detector wavelength and flow rate were 254 nm and 0.8 mL/min, respectively. The oven and injector temperatures were set at 35 °C. The injection volume applied was 100 µL.

### 2.5. Method Validation

Method validation, recovery, limit of detection (LOD), and limit of quantification (LOQ) were determined based on the requirements set in the SANTE document [33]. A series of working standard metsulfuron-methyl solutions ranging from 0.5 mg/L to 5.0 mg/L were injected into the HPLC. The efficiency of the analytical method was evaluated by spiking untreated soil samples at different soil depths of 0–10, 10–20, 20–30, 30–40, and 40–50 cm, and at concentrations of 0.1, 0.5, and 1.0 mg/kg. Six replicates for each concentration were analysed. Next, the calibration curve was plotted vs. peak area to check the linearity and reproducibility. 

### 2.6. Statistical Analysis

The disappearance kinetics of metsulfuron-methyl in the soil was evaluated by plotting the concentration of metsulfuron-methyl residues against time, and the equation of the best fitting curves were estimated by squares of the correlation coefficient (*R*^2^). The dissipation kinetics and half-life of metsulfuron-methyl were calculated from the first-order kinetics equations: *C_t_* = *C*_0_ e^(−*kt*)^ and *t*_½_ = ln 2/*k*, respectively. To get a linear plot, the logarithmic equation was used, ln *C_t_* = ln *C*_0_^(^^−*kt*)^, with variables defined as follows: *C_t_* is the concentration at time *t*, *C*_0_ is the initial concentration, *k* is the rate constant, and *t*_½_ is the half-life.

The experiment design was a randomized complete block with three replications. Data were averaged and subjected to an analysis of variance, and the least significant difference test was calculated at *p* = 0.05 to compare residues means. Analysis of variance and least-squares means were used to evaluate significant differences between treatments’ application rates of metsulfuron-methyl: 15 g a.i/ha versus 30 g a.i/ha. 

## 3. Results

**Figure 1:**
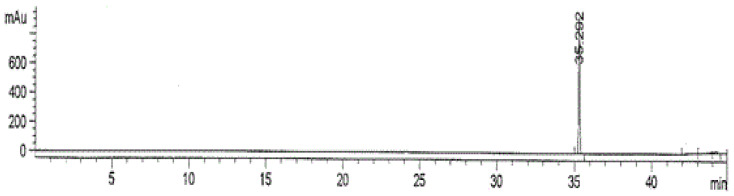
Calibration curve for metsulfuron-methyl.

**Figure 2:**
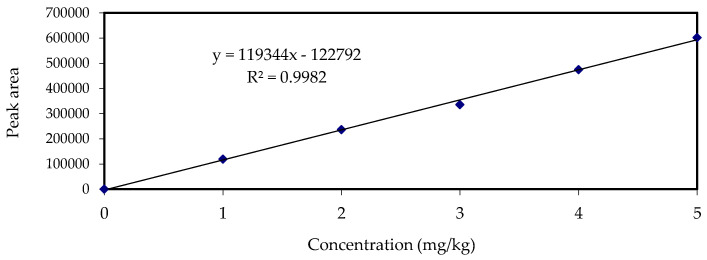
Metsulfuron-methyl standard solution of 1.0 mg/kg.

The analytical method was validated on several parameters, namely linearity, recovery, and limit of detection (LOD). The calibration curve of standard metsulfuron-methyl against the HPLC peak area, obtained using the UV detector, is shown in Figure 1. The linear regression (*R*^2^) was found to be 0.9982, and the equation derived from the calibration area data was *y* = 119344*x*–122792, where *y* was the area of metsulfuron-methyl and *x* was the concentration of metsulfuron-methyl in mg/kg. The retention time of metsulfuron-methyl was 35.292 min (Figure 2). The LOD was found to be 0.05 mg/kg. The recovery of metsulfuron-methyl at the three spiking levels of the concentrations of 0.1, 0.5, and 1.0 mg/kg were 79.68%, 81.55%, and 86.62%, respectively (Table 2). The results of the recovery study at different soil depths, spiked at concentrations of 1.0 mg/kg metsulfuron-methyl, are presented in Table 3. No significant difference (*p* < 0.01) was observed on the percentage of recovery for metsulfuron-methyl extracted from different soil depths.

**Figure 3:**
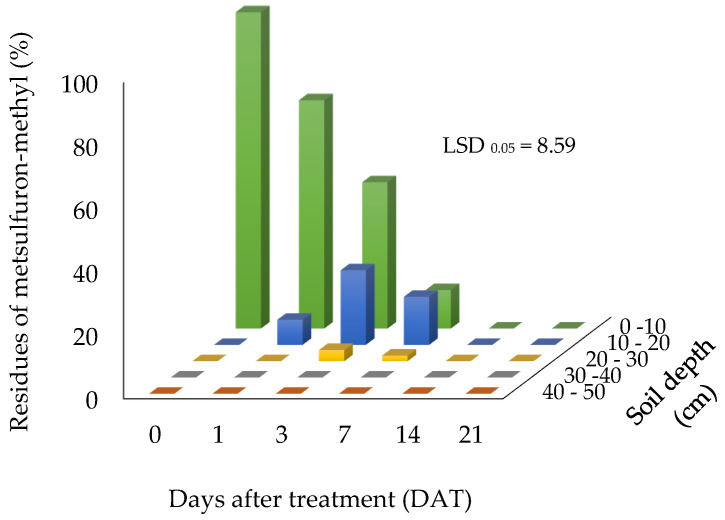
Metsulfuron-methyl residue (%) found at different soil depths when applied at 15 g a.i/ha.

**Figure 4:**
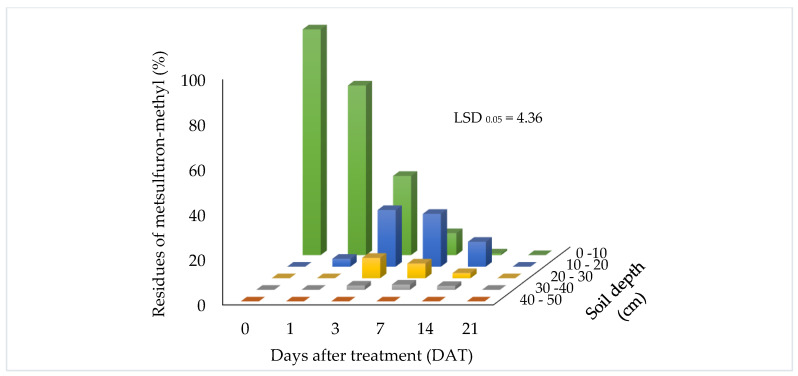
Metsulfuron-methyl residue (%) found at different soil depths when applied at 30 g a.i/ha.

The presence of metsulfuron-methyl residues in the soil profile is illustrated in Figure 3 and Figure 4, at application rates of 15 g a.i/ha and 30 g a.i/ha, respectively. Residues of metsulfuron-methyl were detected at different depths in the soil profile after up to 14 days and 21 days, at application rates of 15 g a.i/ha and 30 g a.i/ha, respectively. Residue levels ranged from 15.94% at 21 days for the application rate at 30 g a.i/ha to 100% at 0 days. Adjustments were done assuming the residue at 0 day was 100%. 

When applied at 15 g a.i/ha, nearly 7.95% of the metsulfuron-methyl moved to the 10–20 cm soil depth after 1 day of application, while 72.23% of the residue still remained at the 0–10 cm soil depth. The remaining approximately 20% of the residue seems to have disappeared in the soil, assuming that it had fully degraded and was therefore undetectable. At 3 and 7 DAT, the residue of metsulfuron-methyl was detected at the 20–30 cm soil depth at 3.56% and 1.78%, respectively. However, no residue was detected at the 30–40 cm and 40–50 cm soil depths throughout the sampling period, demonstrating that metsulfuron-methyl was moderately dynamic in the soil. The percentage of residue detected at all different depths of the soil profile lessened over time. No residue was detected at 14 and 21 DAT. The results show that metsulfuron-methyl residue had degraded to a level that was not detectable at 14 DAT.

A similar trend was observed for the application rate of 30 g a.i/ha. The only difference was that the residue was detected at the 30–40 cm soil depth at 3, 7, and 14 DAT. The concentrations of residue were detected at 1.90%, 2.36%, and 1.74%, respectively. However, no residue was detected at the 40–50 cm soil depth throughout the sampling period. The results demonstrated that the mobility of metsulfuron-methyl increased with higher application rates. These results were in line with studies previous reported [34,35] where the movement of sulfonylureas was found to increase with increased application rate. No residue was detected at 21 DAT. The results show that metsulfuron-methyl residue degraded to a level that was undetectable by 21 DAT. 

**Figure 5:**
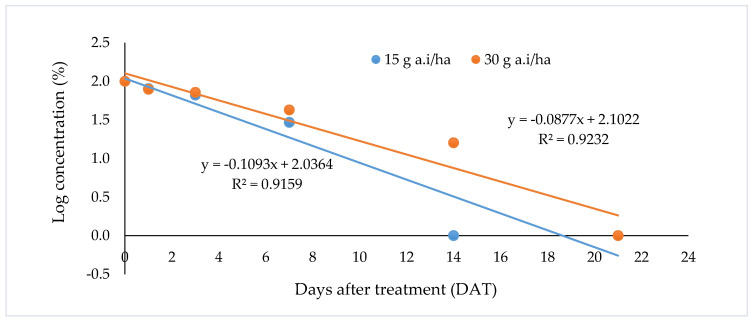
Degradation of metsulfuron-methyl at application rates of 15 g a.i/ha and 30 g a.i/ha.

Degradation of metsulfuron-methyl in the soil when applied at 15 g a.i/ha and 30 g a.i/ha is shown in Figure 5. The data suggests that the degradation of metsulfuron-methyl at both application rates followed first-order kinetics, as both correlation coefficients showed *R*^2^ > 0.9. To calculate the half-life, residue values were logarithmically transformed, and linear fits of the values were plotted against time. Half-lives of 6.3 and 7.9 days were estimated from the first-order kinetics of the degradation process for both application rates (Table 4). Dissipation of ^14^C-phenyl metsulfuron-methyl and ^14^C-triazine metsulfuron-methyl was also found, and followed the first-order kinetics model from our earlier study [5,25]. A similar observation was reported by Ismail and Chong [4], where the half-lives of metsulfuron-methyl in an oil palm field ranged from 7.4 to 9.2 days and 8.2 to 13.4 days when the herbicide was applied at 15 and 30 g a.i/ha, respectively. 

## 4. Discussion

Ismail and Azlizan [26] reported that the half-life of metsulfuron-methyl depends on the soil type, soil temperature, and moisture content. As expected from the known behaviour of metsulfuron-methyl, rapid degradation occurs in the soil at pH and organic contents of 4.13% and 4.03%, respectively. In addition, metsulfuron-methyl has been shown to have fast degradation in soils with high clay content, by increasing the adsorption of metsulfuron-methyl [36]. Therefore, high clay content (40.5%) in the soil may contribute to fast degradation, due to mineralization of metsulfuron-methyl in the soil. The high microbial activity in the oil palm ecosystem might also enhance the degradation of metsulfuron-methyl in the soil. A previous study showed that the DT50 (the time required for 50% of the applied chemical to degrade) values of 14C-phenyl metsulfuron-methyl and 14C-triazine metsulfuron-methyl were 12 days and 14 days, respectively, in non-sterile soil, and 19 days and 23 days in sterile soil [5,25]. These results indicate that degradation of metsulfuron-methyl in the soil is caused by both chemical and microbial processes by soil microorganisms. Furthermore, low concentrations of metsulfuron-methyl could also be due to chemical hydrolysis, the mineralization of metsulfuron-methyl, and the formation of different metabolites, as well as the formation of bound residues [9]. 

**Figure 6:**
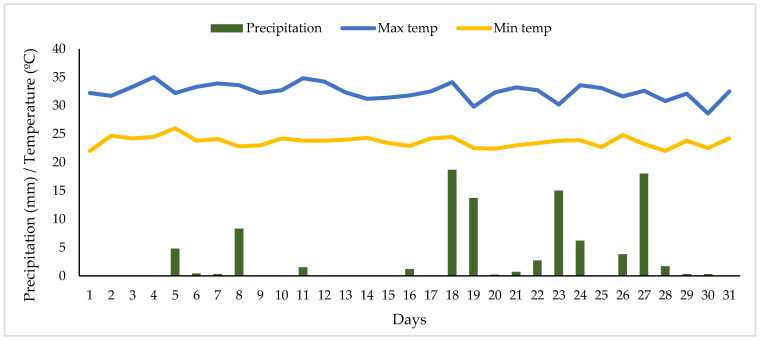
Daily precipitation and temperature recorded during experiment.

Metsulfuron has higher mobility potential in alkaline soils than in acidic soils, as it is more soluble in alkaline conditions [37]. It was also observed that the movement of metsulfuron-methyl through the soil profile was moderately mobile, due to the low pH (pH 4.55) and low organic matter content (4.03%) of the soil. Figure 6 shows daily precipitation and temperature recorded during the experiment. It is important to note that no high rainfall was received throughout the field trial. High rainfall of 18.7, 13.7, 15.0, and 18.0 mm were received on days 18, 19, 23, and 27, respectively. However, no significant effect was observed, as nearly 80% of the metsulfuron-methyl residue was dissipated after 3 DAT. The average annual rainfall recorded is 1800 mm. Daily maximum and minimum air temperature observed were 32.44 °C and 23.63 °C, respectively.

The results of our previous studies suggest that only 10.64% and 19.37% of the applied metsulfuron-methyl (radioactive 14C, labelled metsulfuron-methyl) was found in the leachate when simulated rainfalls of 100 and 200 mL were applied [38]. The above laboratory experiments were conducted using the same type of soil (Bernam soil series). A similar relationship between pH and mobility has been reported for metsulfuron-methyl [4,23,35]. 

## 5. Conclusions

The present study shows that degradation of metsulfuron-methyl under natural conditions at an oil palm plantation was fast, and no residue was detected after 21 DAT when treated with metsulfuron-methyl at 30 g a.i/ha. The dissipation kinetics of metsulfuron-methyl followed first-order kinetics, and the half-life was found to be 6.3 and 7.9 days at application rates of 15 and 30 g a.i/ha, respectively. These results suggested that recurrent application of metsulfuron-methyl in the field has a low risk of residue build-up, due to its rapid degradation. 

## Figures and Tables

**Table 1:** Characteristics of the soil studied.

Soil Type	^1^ CEC (meq/100 g)	Mechanical Analysis (%)				^2^ OM (%)	^3^ OC (%)	pH	Bulk Density (g/cm^3^)
		Clay	Coarse Sand	Fine Sand	Silt				
Loamy clay	26.89	40.50	9.80	18.20	31.50	4.03	2.34	4.13	0.92

^1^ CEC: cation exchange capacity. ^2^ OM: organic matter. ^3^ OC: organic carbon, calculated as OM (%) × 0.58.

**Table 2:** Recovery of metsulfuron-methyl spiked at three concentration levels (*n* = 6).

Concentration (mg/kg)	* Mean Recovery (%)
1.0	86.62 ± 0.65
0.5	81.55 ± 1.05
0.1	79.68 ± 1.12
** LSD_0.05_	2.7256

* Values having the same alphabet are not significantly different at *p* ≤ 0.01, ** LSD0.05, least significant difference at 95% confidence level.

**Table 3:** Recovery of metsulfuron-methyl spiked at 1.0 mg/kg from different soil depths (*n* = 6).

Soil Depth (cm)	* Mean Recovery (%)
0–10	85.32 ± 0.82
10–20	84.55 ± 1.10
20–30	85.12 ± 0.72
30–40	84.59 ± 0.43
40–50	86.33 ± 1.21
** LSD_0.05_	2.3681

* Values having the same alphabet are not significantly different at *p* ≤ 0.01. ** LSD0.05, least significant difference at 95% confidence level.

**Table 4:** First-order rate constant (*K*), half-life (*t*_1/2_), and correlation coefficient (*R*^2^) of metsulfuron-methyl at two application rates.

Application Rate (g a.i/ha)	*R* ^2^	*K* (Days^−1^)	*t*_1/2_ (Days)
15	0.9159	0.1093	6.3
30	0.9202	0.0877	7.9
